# Local Activation of the Alternative Pathway of Complement System in Mycotic Keratitis Patient Tear

**DOI:** 10.3389/fcimb.2020.00205

**Published:** 2020-05-06

**Authors:** Mohammed Razeeth Shait Mohammed, Sandhya Krishnan, Rabbind Singh Amrathlal, Jeya Maheshwari Jayapal, Venkatesh Prajna Namperumalsamy, Lalitha Prajna, Dharmalingam Kuppamuthu

**Affiliations:** ^1^Department of Proteomics, Aravind Medical Research Foundation, Dr. G. Venkataswamy Eye Research Institute, Aravind Eye Care System, Madurai, India; ^2^Department of Microbiology, Aravind Medical Research Foundation, Dr. G. Venkataswamy Eye Research Institute, Aravind Eye Care System, Madurai, India; ^3^Cornea Clinic, Aravind Eye Hospital, Aravind Eye Care System, Madurai, India; ^4^Department of Ocular Microbiology, Aravind Eye Hospital, Aravind Eye Care System, Madurai, India

**Keywords:** fungal keratitis, *A. flavus*, complement alternate pathway, C3b, factor H and factor I

## Abstract

*Aspergillus flavus and Fusarium solani* are the predominant causative agents of mycotic keratitis in the tropical part of the world. Tear proteins play a major role in the innate immune response against these fungal infections as has been shown by the presence of complement proteins and neutrophil extracellular trap proteins in keratitis patients tear. In this study, we established the presence of the components of the alternate pathway of complement system and their functional state in the tear film of mycotic keratitis patients. The complement proteins namely, C3 and CFH were found only in the open-eye tear of patients but not in control individuals. *In vitro* analysis showed binding of purified C3b and CFH to fungal spores, which confirmed that the spores can provide a foreign surface for forming the complement complex. Analysis of spore bound tear proteins by mass spectrometry exhibited the presence of known proteins of the alternate pathway complement cascade in keratitis patient tear. Hemolytic assay using rabbit RBC confirmed the presence of a functional alternate pathway of complement cascade in the tear proteome of the patients. The presence of negative regulators, CFH and CFI, in the patient tear indicate that the complement activity is tightly regulated during fungal infection. Mass spectrometry data show vitronectin and clusterin, two known inhibitors of the membrane attack complex only in the patient tear. These data demonstrate the activation of the alternate pathway of complement cascade during the early stages of infection. Interestingly, the production of multiple negative regulators of complement cascade implies the pathogen can effectively evade the host complement system during infection.

## Introduction

Fungal keratitis is one of the sight-threatening corneal infections, which develops in immunocompetent individuals also (Selvam et al., 2015). Mycotic keratitis is more widespread in India and other tropical parts of the world (Erie et al., 1993; Mohammed et al., 2019b). Tear film plays a crucial role in immune defense against the invading microbes and the presence of most of the proteins involved in the alternate pathway of complement cascade has been demonstrated in patient tear (Ananthi et al., 2013; Kandhavelu et al., 2017). Previous studies showed the presence of several innate immune response associated proteins such as NET proteins, wound healing proteins and complement proteins in patient tear (Kandhavelu et al., 2017). Activation of the alternate pathway requires appropriate foreign surface for binding of the complement proteins and their subsequent activation. The complement system is controlled by negative regulators including factor H-like protein 1 (FHL-1), factor H (CFH), and complement factor I (CFI). CFH is the key regulator of C3b amplification and prevents non-specific damage to host cells (Ram et al., 1998; Zipfel and Skerka, 1999; Behnsen et al., 2008; Schmidt et al., 2016). CFH accelerates irreversible decay of C3bBb by displacing Bb, as well as complement factor I (CFI) mediated cleavage of C3b (Kuhn and Zipfel, 1996, Pangburn et al., 1977; Irmscher et al., 2018), yielding iC3b that cannot bind CFB. Subsequently iC3b cleavage ultimately yields C3d, and these regulatory functions prevent host damage by terminating the complement cascade. Among the fungi studied, *Candida albicans* (Meri et al., 2002) and *A. fumigatus* (Kozel et al., 1989; Johnsson et al., 1998) are shown to bind complement regulators to their surfaces leading to immune evasion due to the down regulation of complement activation. The presence of complement proteins C1q, C3, CFB, C4, C5, and C9 have been shown in closed- eye tears. However, only C3, CFB, and C4 are found in open-eye tears (Willcox et al., 1997). These proteins in the tear are shown to be active functionally. Our previous studies have shown the presence of several complement proteins in the tear proteome of keratitis patients (Kandhavelu et al., 2017). We also showed the presence of negative regulators namely, CFH, vitronectin and clusterin (inhibitors of the membrane attack complex), and lactoferrin (acts on soluble C3) (Kandhavelu et al., 2017). Previous reports clearly showed lactoferrin, an abundant protein found in human tear, inhibit the classical pathway of complement cascade but not the alternative pathway (Kievjts and Kijlstra, 1985).

The aim of the present work was to confirm the presence of alternative pathway of complement proteins and the complement regulatory proteins in the tear film of keratitis patients and to show their functional competence.

## Materials and Methods

### Tear Protein Samples, *A. flavus* Strains and Their Growth Conditions

*Aspergillus flavus* strain CI1123 used in this study has been described previously (Selvam et al., 2015; Mohammed et al., 2019b). Conidia were harvested using 0.05% (v/v) Tween 20 in PBS (pH 7.2), filtered, counted using a Neubauer counting chamber and the spore suspension was stored in 20% glycerol at −80°C. For liquid culture, 50 ml of Czapek Dox broth (Himedia) was inoculated with conidia and incubated at 30°C for 2 h to obtain swollen spores.

This study was approved by the Institutional Ethical committee of Aravind Eye Hospital Madurai and informed consent was obtained from all study participants. Tear samples were collected from patients and uninfected age-matched controls as described previously (Kandhavelu et al., 2017). The method used for tear collection has been optimized to avoid contamination of cells from corneal epithelial layer. All the samples used in this study were open-tear samples. We did not find any significant variation in the total volume of tear collected from individuals from both groups.

### Identification of CFH and C3b in Patient Tear

Tear samples from keratitis patients were pooled and 12 μg of tear proteins were subjected to sodium dodecyl sulfate-polyacrylamide gel electrophoresis (SDS-PAGE). Proteins were transferred onto a nitrocellulose (NC) membrane using a semi dry blotter (Thermo Scientific). The NC membrane was equilibrated with Towbin transfer buffer [39 mM glycine, 48 mM Tris-Cl, pH 7.5, and 20% methanol] and blocked with 5% skimmed milk powder in Tween 20-Tris buffered saline (TBS-T) to prevent non-specific binding. Immuno detection was performed by incubating the membrane overnight with rabbit anti-human Complement factor H antibody (H-300; SC33156, Santa Cruz Biotechnology) diluted 1:5,000 in TBS containing 0.1% skim milk powder to detect CFH and C3b was detected using rabbit monoclonal anti-C3 antibody (EPR2988 [Recombinant rabbit monoclonal antibody raised using synthetic peptide spanning human C3dg region (aa 1,200–1,300) of C3 protein], Abcam). This antibody can detect the breakdown products of C3 alpha chain, namely C3α, C3bα', iC3bα'1, C3dg, and C3d. After three washes with TBS-T and TBS, the membrane was incubated with goat anti-rabbit IgG HRP conjugate (Abcam) diluted 1:5,000 in TBS containing 0.1% skim milk powder at RT for 45 min. After three washes with TBS-T, the membrane was developed using 3, 3′-diaminobenzidine chromogenic substrate. The color development was stopped by rinsing the membrane in Milli-Q water. The membrane was then air-dried and imaged using Gel Doc^TM^ (Bio-Rad Laboratories). In all these experiments, in the absence of a tear protein that is unaltered in level for use as a loading control, extra care was taken to quantify the exact concentration of tear proteins using Bradford assay followed by a check gel confirmation. Rainbow protein marker allowed the confirmation of transfer efficiency in western blot assay.

### Binding Assay to Determine CFH and Spore Interaction

*Aspergillus flavus* swollen spores (2 × 10^8^), were suspended in 150 μl of binding buffer (100 mM NaCl, 50 mM Tris [pH 7.4]) and incubated with 2 μg of CFH (Merck Millipore) at 37°C for 1 h with mixing (4 rpm). At the end of incubation period, the conidia were washed five times with one ml of wash buffer (100 mM NaCl, 50 mM Tris, 0.05% Tween 20 [pH 7.4]) (Behnsen et al., 2008) and, the bound proteins were eluted by boiling spores in 50 μl of 1X Laemmli buffer for 5 min. After removing spores by centrifugation, the proteins in the supernatant were resolved by SDS-PAGE followed by western blot using anti-CFH antibody.

### Immunofluorescence Assays

*Aspergillus flavus* swollen spores (1 × 10^8^) were mixed with 10 μg of factor H pure protein and incubated at 37°C for 1 h. The conidia were washed three times with ice-cold PBS (0.03 M phosphate, 0.15 M NaCl [pH 7.2]), and suspended in 2% BSA in PBS to avoid non-specific binding and incubated for 30 min at 37°C. Factor H bound conidia were incubated with anti-factor H primary antibody for 1 h at 37°C (1:200 dilution), followed by three washes in PBS and then mixed with the goat anti-rabbit IgG-FITC secondary antibody [F9887, Sigma, dilution of 1:400 in 2% (w/v) BSA-PBS] for 1 h. The conidia were washed once with PBS and mixed with 10 μg/ml calcofluor for 30 min at RT. Excess calcofluor was removed by washing and the stained conidia were examined using the Leica Live confocal microscope. The experiment was repeated as above for C3b immunofluorescence detection.

### Assay of Binding of Tear Proteins to Spores

*Aspergillus flavus* swollen spores (2 × 10^8^) were mixed with 100 μg of infected tear proteins in 150 μl of binding buffer and the mixture was incubated for 1 h at 37°C. Unbound proteins were removed by washing the conidia five times with one ml of wash buffer (100 mM NaCl, 50 mM Tris, 0.05% Tween 20 [pH 7.4]). Proteins bound to conidia were eluted by boiling in 50 μl of 1X Laemmli buffer. The supernatant containing eluted proteins were collected by centrifugation and used for SDS-PAGE separation and probed with anti-C3 antibody or anti-CFH antibody in western blots.

### Heparin Competition Experiments

Heparin (heparin sodium salt, Himedia, TC138; 5,000 IU/ml) was mixed with 5 μg of CFH and incubated for 30 min at 37°C. The complex was added to swollen conidia (2 × 10^8^) and incubated for 1 h with constant mixing. Washing and elution of the bound CFH was examined as described above.

### Assay of Cleavage of C3b by CFI

Swollen conidia (2 × 10^8^) were incubated with 100 μg of *A. flavus* keratitis patient tear for 1 h at 37°C on a shaker with or without the addition of 1.5 μg of CFI (Merck Millipore). At the end of the incubation period, conidia were separated by centrifugation and the supernatant was taken for SDS-PAGE and western blot analysis using anti-C3 antibody.

### Spore Surface Bound CFH Accelerates Proteolysis of C3b by CFI

Swollen conidia (2 × 10^8^) were incubated with purified CFH (5 μg) for 1 h at 37°C on a shaker. The mixture was washed five times with washing buffer and resuspended in 150 μl of binding buffer. Three micrograms of C3b (Merck Millipore) and 1.5 μg of CFI were added. The conidia were incubated for 1 h at 37°C, the conidia were removed by centrifugation, and the released cleavage products were separated by SDS-PAGE and analyzed by immunoblotting.

### MS Analysis of Spore Bound Tear Proteins

Swollen conidia (2 × 10^8^) were incubated with 100 μg of tear from *A. flavus* keratitis patient (intermediate stage) or healthy person and the bound proteins were eluted by boiling in 50 μl of 1X Laemmli buffer for 5 min as discussed above. Proteins in the supernatant were fractionated on a 12% SDS-PAGE. Electrophoresis was stopped when the tracking dye reached one cm into the separating gel. After staining with coomassie blue, a single band was cut and processed for in-gel tryptic digestion and mass spectrometry as described previously by Kandhavelu et al. (2017) with minor modifications. In brief, gel pieces were destained with repeated washes with 25 mM ammonium bicarbonate in 50% acetonitrile. The gel pieces were dehydrated using 100% acetonitrile followed by reduction of disulfide bridges using 50 μl of 10 mM DTT in 25 mM ammonium bicarbonate for 45 min at 55°C and alkylated with 55 mM IAA in 25 mM ammonium bicarbonate. After reduction and alkylation, gel pieces were washed three times with 100 μl of 100 mM ammonium bicarbonate, and dehydrated using 100% acetonitrile. Dehydrated gel pieces were dried under vacuum and rehydrated for 30 min on ice with 600 ng of trypsin (Invitrogen). Tryptic peptides were purified using C18 tips and analyzed using Thermo Easy nLC 1000 coupled with Orbitrap Velos Pro mass spectrometer (Thermo, USA) and MS parameters are discussed in detail in our previous paper (Mohammed et al., 2019a).

### Data Processing

All MS/MS raw data for experimental and biological replicates acquired from Orbitrap Velos Pro Mass Spectrometer were analyzed by Proteome Discoverer v1.4 using Mascot and the inbuilt SequestHT algorithm. Both SequestHT and Mascot was set up to search against human proteome database from UniProt (141,139 entries) with parameters of peptide tolerance of 10 PPM with two missed cleavages. Carbamidomethylation was given as fixed modification and methionine oxidation, N-terminal acetylation and phosphorylation (S, T, Y) as variable modifications (Selvam et al., 2015; Mohammed et al., 2019a).

### Functional Assay for Alternative Pathway of Complement System

Functional assay using rabbit blood was done as described previously (Sohn et al., 2000) with minor modification. Five ml of rabbit blood was mixed with an equal volume of Alsever solution and kept on ice for 5–10 min. RBCs were washed twice using DGHB buffer and suspended in fresh DGHB and kept at 4°C. Cells were counted using a Neubauer counting chamber. Hemolytic assay was performed by diluting human serum from a healthy person in DGHB buffer at ratio of 1:4 and 1:6 or with different concentrations (200 μg, 400 μg, 600 μg, 800 μg, 2 mg) of control/keratitis tear. Tear or serum was mixed with 1 × 10^8^ rabbit RBCs and incubated for 60 min at 37°C. Reaction was terminated by adding 1.2 ml of ice-cold 0.15 M NaCl. After centrifugation (1,250 x g) for 10 min at 4°C, the clear supernatant was used for optical density measurement at 412 nm using spectrophotometer (Evolution UV- VIS Thermo Fisher). Optical density of the control experiment, where water was substituted for proteins, was taken as 100% lysis (Sohn et al., 2000).

## Results

### C3 Protein and Its Cleavage Products in Keratitis Patient's Tear

**Figure 1:**
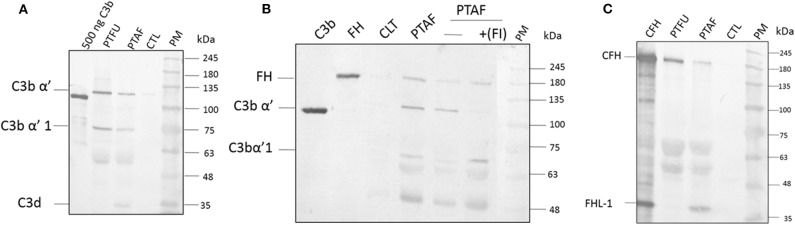
Complement C3, factor H and factor I are present in keratitis patient tear and are functional. **(A)** C3 protein and its cleavage products in keratitis patient tear. Twelve microgram of tear proteins was separated on a 12% SDS-PAGE and probed with anti-C3 antibody as described in materials and methods. The monoclonal antibody used recognized the C3dg portion of the C3 protein. Lanes 1, Purified C3b (500 ng); 2, *Fusarium* keratitis patient tear (PTFU); 3, *A. flavus* keratitis patient tear (PTAF); 4, normal person tear (CTL); 5, protein marker (PM). **(B)** Alpha chain of C3 is cleaved by patient tear and externally added CFI can accelerate this cleavage. *A. flavus* swollen conidia (2 × 10^8^) were incubated with 50 μg of keratitis patient tear in the presence or absence of Factor I (1.5 μg) and the supernatant was separated in a SDS-PAGE and transferred to NC membrane. The membrane was probed with a mix of anti-FH and anti-C3 antibodies. Lanes 1, Purified C3b; 2, Purified CFH; 3, Control tear; 4, *A. flavus* keratitis patient tear (PFTA); 5, *A. flavus* keratitis patient tear without factor I (FI); 6, *A. flavus* keratitis patient tear mixed with FI; 7, protein marker (PM). **(C)** Complement factor H in keratitis patient tear. Twelve microgram of tear protein was separated on a 12% SDS page and probed with anti-CFH antibody in western blots as described in materials and methods. Lanes 1, Purified CFH (500 ng); 2, *Fusarium* keratitis patient tear (PTFU); 3, *A. flavus* keratitis patient tear (PTAF); 4, normal person tear (CTL); 5, protein marker (PM).

Proteins from tear films were fractionated on acrylamide gels and, the presence of C3 and its processed products were examined using a monoclonal antibody that recognizes an epitope in the C3dg portion of C3-alpha chain. This antibody was shown to recognize C3 (α, 120 kDa), C3b (α', 110 kDa), iC3b (α'1,68 kDa), C3dg (41 kDa), and, C3d (31 kDa). Data in Figure 1A show the presence of the C3 alpha chain belonging to uncleaved C3, migrating as a 120 kDa band as well as the cleaved forms of C3. The presence of uncleaved form of C3 was previously reported by us (Kandhavelu et al., 2017) and, our current data confirm this finding. In the control sample, only a faint band migrating at the 120 kDa region could be detected compared to the patient's tear, indicating the induction of C3 upon fungal infection. The presence of the alpha fragment of iC3b in both *Fusarium* and *A. flavus* keratitis patient tear implied the activation of complement pathway. Unlike the tear from *A. flavus* keratitis patients, the quantity of C3 and its cleaved products were slightly higher in *Fusarium* keratitis patient tear, even though same amount of total tear proteins were loaded in each track (Figure 1A). C3 and its cleavage products were significantly reduced in control tear.

### C3 Convertase Is Cleaved in Patient Tear

The presence of CFH well as the formation of iC3b (Figure 1B) indicated the cleavage of C3 in patients' tear, presumably mediated by complement factor I (CFI). Data in Figure 1B show the presence of CFH and C3 in *A. flavus* keratitis tear. Previous studies using *A. fumigatus* spores showed that the alternative pathway of complement can be assembled on the spore surface (Behnsen et al., 2008). Therefore, we examined the formation of C3 convertase on the surface of *A. flavus* spores. Swollen spores when added to tear proteins led to the reduction in the amount of C3 in the tear samples (compare the 120 kDa C3 protein band in lanes 4 and 5 of Figure 1B). Further, the addition of purified CFI led to complete cleavage of C3, which implied the reduced cleavage seen in patient tear is due to the limitation in the amount of CFI. Further experiments are needed to confirm the absence of other inhibitors of C3 cleavage.

### Identification of Complement Factor H in Tear Samples

Previous mass spectrometry data (Kandhavelu et al., 2017) show the presence of CFH, the major negative regulatory factor of the alternative complement pathway, in keratitis patient's tear. This data was further confirmed in this study by western blot analysis of CFH in the tear sample from the patients and control (Figure 1C). The protein band corresponding to 180 kDa co-migrating with purified CFH showed the presence of CFH in keratitis patients' tear but not in control tear. The anti-CFH antibody recognized an additional protein migrating as a 37 kDa band even in purified CFH sample lane. This protein has been shown to be factor H like (FHL) protein (unpublished results) and has been identified from keratitis patient tear as well (see later). FHL, however, could not be found in *Fusarium* keratitis patient tear. Two other cross-reacting proteins detected have not been identified yet. The presence of CFH implies the negative regulation of the alternative pathway since CFH catalyzes CFI mediated cleavage of C3b, leading to the inhibition of C3 convertase formation.

### C3b and CFH Bind Efficiently to the Conidial Surface

**Figure 2:**
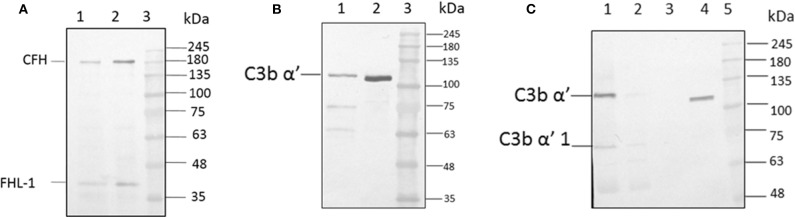
C3b and CFH binds to conidial surface. **(A)** *A. flavus* conidia were incubated with 2.0 μg factor H, washed and eluted the surace bound proteins, which were separated by SDS-PAGE, transferred to a NC membrane and probed with anti-CFH antibody. Lanes 1, Eluate of 2.0 μg of CFH bound to the spore; 2, 200 ng of pure protein CFH; 3, protein marker (PM). **(B)** *A. flavus* conidia (2 × 10^8^) were incubated with purified C3b (5 μg). After extensive washing, bound proteins were eluted with 1X Laemmli buffer. Proteins in eluate were separated in a SDS-PAGE, transferred to a NC membrane and probed with anti-C3b antibody. Lanes 1, Eluate of 5 μg of C3b bound to the spore; 2, 500 ng of pure protein C3b; 3, protein marker (PM). **(C)** Binding of C3b in keratitis patient tear to conidial surface. *A. flavus* conidia were incubated with purified 50 μg of *A. flavus* keratitis patient tear. After extensive washing, bound proteins were eluted with 1X Laemmli buffer. Proteins in the eluate were separated in a SDS-PAGE, transferred to a NC membrane and probed with anti-C3b antibody. Lanes 1, 10 μg of keratitis patient tear; 2, unbound proteins of keratitis patient tear; 3, wash fraction; 4, eluate of patient tear proteins bound to the spore surface; 5, protein marker (PM).

**Figure 3:**
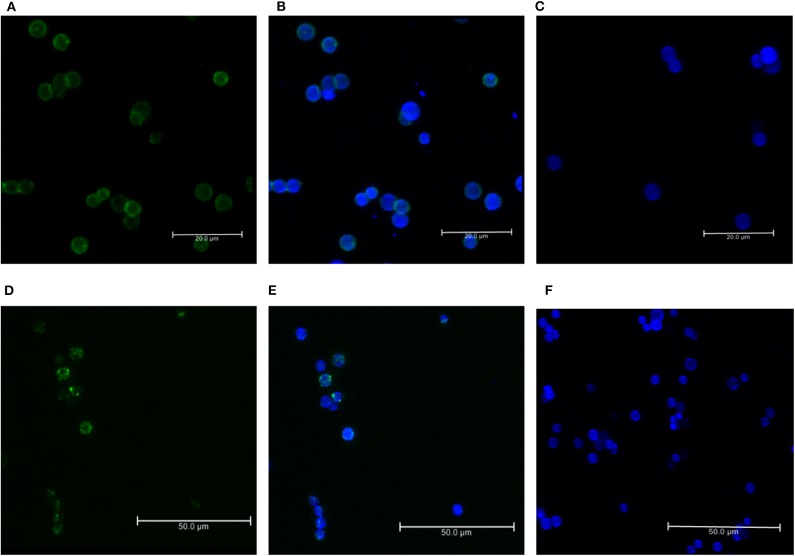
Immunofluorescence of C3b and CFH binds to conidial surface. Binding of factor H and C3b to *A. flavus* conidia. Conidia were incubated with purified factor H and C3b separately and binding was visualized by immunofluorescence with rabbit anti-CFH antibody and an Alexa 488-coupled secondary antibody (green). The cell wall was stained with calcofluor (blue). **(A)** Immunofluorescence of conidia labeled with C3b (FITC-green). **(B)** Merged image of conidia labeled with C3b-FITC and calcofluor. **(C)** Negative control without the C3b. **(D)** Immunofluorescence of conidia labeled with CFH (FITC-green). **(E)** Merged image of conidia labeled with CFH-FITC and calcofluor. **(F)** Negative control without C3b.

For the formation of functional complement components, C3 and C3b should bind to the surface of spores of *A. flavus* and the cleavage of bound C3b by CFH also depends on the binding of CFH to surface. In order to examine this, conidia were incubated with purified CFH or C3b and at the end of the incubation period, the conidia were washed extensively, and the proteins bound to the spores were eluted and examined. Proteins in the eluted fraction were separated by SDS-PAGE and analyzed by Western blotting using appropriate antibodies. Figures 2A,B shows the presence of CFH and C3b, respectively, in the bound fraction. In both cases, the binding is nearly complete since the wash fractions did not have any proteins (data not shown). Binding of CFH and C3b to *A. flavus* was also analyzed by immunofluorescence as described in materials and methods section. Figure 3 shows the fluorescence of antibody bound to *A. flavus* spores pre-incubated with C3b and CFH but not in untreated spores. These experiments confirm that the *A. flavus* spore surface can act as an activating surface for the assembly of the C3 convertase as well as its inhibitory components (Figure 2C).

### Inhibition of C3b and CFH Binding to Spore Surface by Heparin

**Figure 4:**
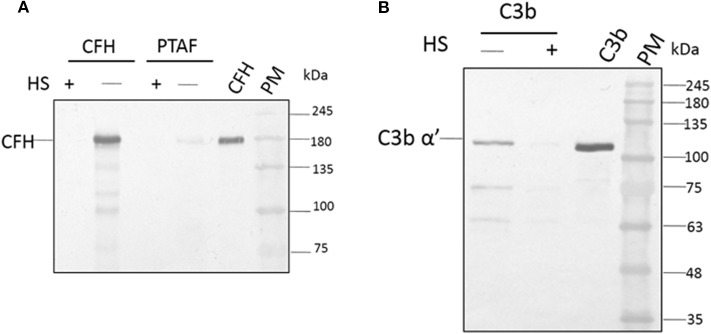
Heparin inhibits the binding of factor H to conidial surfaces. **(A)** *A. flavus* swollen conidia (2 × 10^8^) were incubated with purified factor H (5 μg) in the presence or absence of 5,000 IU of heparin sulfate (HS) or with 100μg of *A. flavus* infected tear in the presence of heparin (5,000 IU). Bound proteins were eluted with a 1X Laemmli buffer, eluate fractions were separated on a SDS-PAGE and western blot analysis performed using anti-CFH antibody. Two hundred nanogram of purified factor H was used as a control. Lanes 1, Eluate of 5,000 IU of HS with 5 μg CFH; 2, eluate of 5 μg CFH bound to spores; 3, eluate of 5,000 IU of HS with 100μg *A. flavus* infected tear; 4, 12 μg of *A. flavus* keratitis patient tear; 5, purified CFH 200 ng; 6, protein marker (PM). **(B)** *A. flavus* swollen conidia (2 × 10^8^) were incubated with purified C3b (5 μg) in the presence and absence of 5,000 IU of heparin sulfate (HS). After extensive washing, bound proteins were eluted with 1X Laemmli buffer. Proteins in eluate were separated in a SDS-PAGE, transferred to a NC membrane and probed with anti-C3b antibody. Lanes 1, eluate of 5 μg of C3 bound to spores; 2, eluate of 5,000 IU of HS with 5 μg C3b; 3, purified C3b; 4, protein marker (PM).

Among the SCR domains of CFH, SCR 7, SCR 9, SCR 13, and SCR 20 are responsible for binding heparin (Behnsen et al., 2008). Since these same sites are also involved in the binding of CFH to the microbial surface, we examined the role of these sites in the binding of *A. flavus* spores. At a concentration of 5,000 IU/ml, heparin completely inhibited the attachment of CFH to conidia, as demonstrated by western blot analysis (Figure 4A, compare lane 1 and 2). Binding of CrFH in keratitis patients' tear to the *A. flavus* spore surface was inhibited when the tear was pretreated with heparin. In a similar experiment, C3b was pre-incubated with heparin (5,000 IU/ml) and the binding of C3b to spores was shown to be inhibited (Figure 4B). The actual mechanism of C3b binding to spore surface is unknown, however, the above data imply that the heparin-binding sites in C3b are the ones involved in spore binding.

### Heparin Accelerated Inhibition of CFH Mediated Cleavage of C3b by CFI

**Figure 5:**
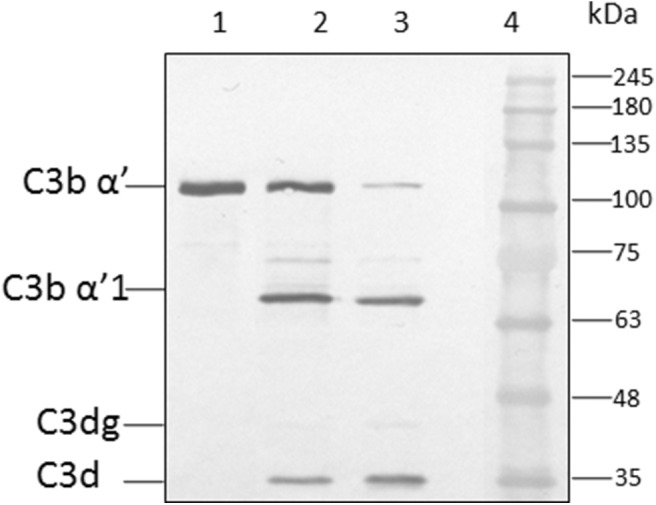
Co-factor assay. Conidia of *A. flavus* were incubated with purified factor H in the presence or absence of 5,000 IU of heparin sulfate (HS). After extensive washing, both factor I and C3b were added. After incubation for 1 h, the supernatant was separated and proteins resolved by SDS-PAGE, transferred to a NC membrane and probed with anti-C3 antibody. The positions of the C3bα' and it's cleaved forms are indicated. Lanes 1, purified C3b control (500 ng); 2, supernatant of C3b and FI after incubation with 5,000 UI of Heparin sulfate and 5 μg CFH; 3, supernatant of C3b and FI after incubation with 5 μg CFH on conidia; 4, protein marker (PM).

Earlier study show the CFH binding to foreign surface leads to the CFH mediated CFI cleavage of C3b (Behnsen et al., 2008). In order to examine the effect of heparin on CFH mediated cleavage of C3b through CFI, conidia were incubated with CFH prior to the addition of heparin (5,000 IU/ml) and after extensive washing, purified CFI and C3b were added. After incubation, the bound proteins were eluted and separated by SDS-PAGE. The proteolytic cleavage of α'chain of C3b was assayed by western blotting. In the presence of CFI and C3b, CFH-coated conidia accelerated the cleavage of C3b as expected (Figure 5, lane 3). However, the addition of heparin inhibits CFH (Figure 5, lane 2) mediated cleavage confirming the inhibition of C3b binding to spores.

### Assembly of Complement Proteins in Tear on the Spore Surface

Previous studies show the presence of several complement proteins in patient tear but not in control tear. In order to examine the binding of complement proteins to spores, tear proteins were incubated with conidia and the bound proteins were eluted and analyzed using mass spectrometry as described under materials and methods. Table 1A shows the list of complement proteins in keratitis patients' tear bound to *A. flavus* spores. In addition to the complement proteins, vitronectin, and clusterin that are inhibitors of MAC complex formation, were also found in patient tear (Table 1B). We also found proteins involved in neutrophil extracellular trap formation along with lactoferrin, a protein that regulates complement activity in the spore bound protein fraction.

**Table 1:** Hundred microgram of *A. flavus* keratitis patient tear was incubated with *A. flavus* conidia for 60 min at 37°C.

**(A) IDENTIFICATION OF KERATITIS PATIENT TEAR PROTEINS BOUND TO *A. flavus* CONIDIAL SURFACE**			
**Uniprot ID**.	**Protein description**	**Number of unique peptides**	**PSMs**
P01024	Complement C3	12	42
A8K5T0	cDNA FLJ75416, highly similar to CFH	9	28
F5GXS0	Complement C4-B	3	10
P02747	Complement C1q subunit C	3	12
D6RA08	Complement C1 subunit B	2	4
F8WCZ6	Complement C1s	2	4
B0UXW4	Complement factor B	3	10
B4E1B0	cDNA FLJ54318, highly similar to Complement C1r	2	3
B1AKG0	Complement factor H-related protein 1	1	2
P01625	Ig kappa chain V-IV region Len	2	8
B7Z553	cDNA FLJ51266, Vitronectin OS	1	14
P10909-4	Isoform 4 of Clusterin	6	24
P80188	Neutrophil gelatinase-associated lipocalin	2	6
B0QY04	Neutrophil cytosol factor 4	1	4
P59665	Neutrophil defensin 1	1	1
B1ALB7	Neutrophil cytosol factor 2	1	1
B3VMW0	Lactoferrin	31	717
B2MV14	Truncated lactoferrin	22	609
B3KSL2	cDNA FLJ36533, highly similar to lactotransferrin	31	769
P08311	Cathepsin G	3	9
P05164-2	Isoform H14 of Myeloperoxidase	4	18
B4DNT5	Proteinase 3	1	2
P61626	Lysozyme C	11	764
**(B) COMPARISON OF THE SPORE BOUND PROTEINS OF CONTROL AND PATIENT TEAR FILM**			
**Uniprot ID**	**Protein description**	**Control tear (PSMs)**	**Patient tear (PSMs)**
E7ER44	Kaliocin-1	1,676	–
B3VMWO	Lactoferrin	1,663	674
B3KSL2	cDNA FLJ36533 highly similar to lactotransferrin	–	642
Q5EK51	Lactoferrin	1,652	–
Q2TUW9	Lactoferrin	1,610	–
P02787	Sero transferrin	–	136
B2MV14	Truncated Lactoferrin	1,410	475
H6VRF8	Keratin 1	267	307
Q6MZV7	Uncharacterized protein DKFZp686C11235	–	165
P13645	Keratin, type I cytoskeletal 10	178	222
P02647	Apolipoprotein A-I	–	83
P35908	Keratin, type II cytoskeletal 2 epidermal	99	168
P35527	Keratin, type I cytoskeletal 9	105	126
F6KPG5	Albumin	77	1,220
P61626	Lysozyme C	652	488
P12273	Prolactin-inducible protein	–	49
A8K008	cDNA FLJ78387	–	156
P05109	Protein S100-A8	–	55
Q6GMX6	IGH protein	–	155
B4E335	cDNA FLJ52842, highly similar to Actin, cytoplasmic 1	–	–
Q6MZQ6	Uncharacterized protein DKFZp686G11190	–	144
Q6N096	Uncharacterized protein DKFZp686I15196	–	146
P01859	Ig gamma-2 chain c region	–	114
P01860	Ig gamma-3 chain c region	–	98
Q6MZU6	Putative uncharacterized protein DKFZp686C15213	–	111
B4DW52	cDNA FLJ55253, highly similar to Actin, cytoplasmic 1	–	99
Q0KKI6	Immunoglobulin light chain (fragment)	–	98
P31025	Lipocalin-1	93	197
P25311	Zinc-alpha-2-glycoprotein	33	18

*After extensive washing, bound proteins were eluted by using sample buffer. Eluate fraction was processed for shot-gun MS sample preparation. Raw files were analyzed in Proteome Discoverer. Comparison of complement protein bound to conidia present in infection tear is given in **(A,B)** shows comparison of bound proteins using control and infected tear in the spore binding assay*.

### Demonstration of Functional Competence of the Alternate Pathway in the Keratitis Patient Tear

**Figure 6:**
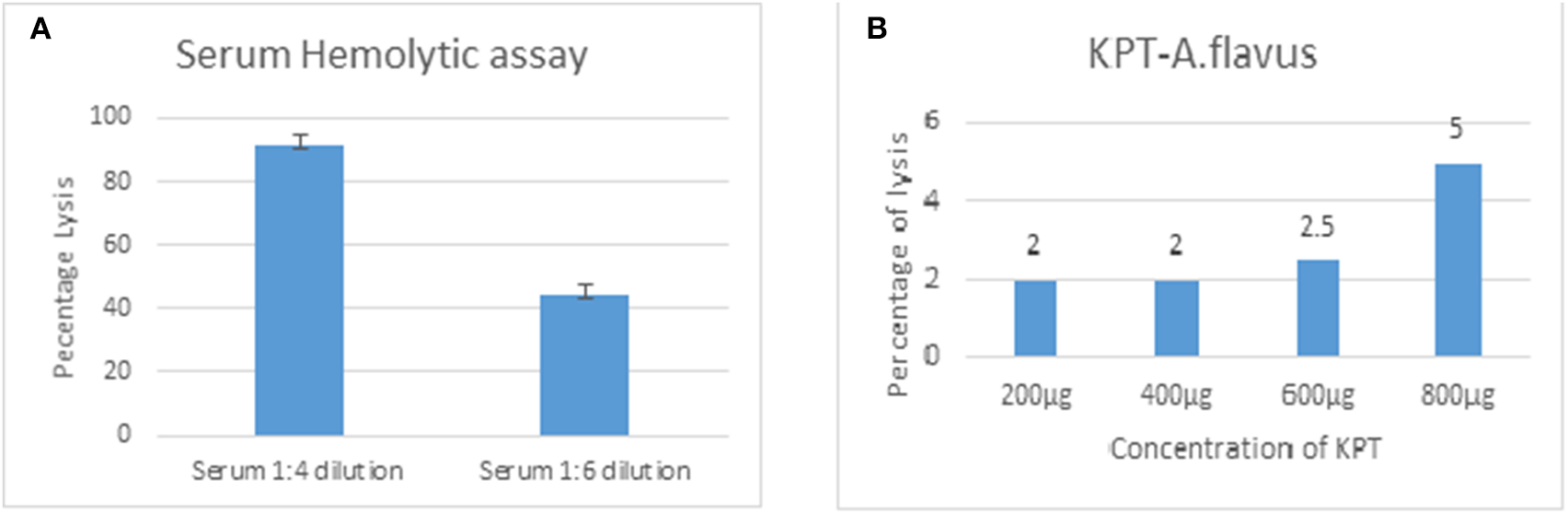
Functional assay of complement proteins. Different concentrations of human serum **(A)** or *A. flavus* keratitis patient tear **(B)** was added to 1 × 10^8^ to rabbit RBCs and incubated in DGHB buffer for 60 min at 37°C. After centrifugation, the supernatant was collected and the absorbance measured at 412 nm.

Rabbit erythrocytes are not protected from CFH mediated inhibition of hemolysis when mixed with human serum (Herbert et al., 2015). This assay is useful to demonstrate the presence of all the complement components and the formation of the final membrane attack complex that lyses intact cells. Data in Figure 6 show 55% hemolysis of rabbit RBCs in six-fold diluted serum. RBC hemolysis could be demonstrated with tear proteins from keratitis patients only at higher concentrations. The presence of lower amount of complement components as well as inhibitors of the complement cascade could be the reason for the decreased hemolysis in tear film compared to serum. Unlike the patient tear, tear from controls did not show any hemolytic activity even at a high concentration of 2 mg.

## Discussion

The significant findings of the current work are, one, fungal infection induces all components of the alternative pathway of the complement cascade, and two, these complement proteins can form a functional membrane attack complex.

This study confirms the previous mass spectrometry study (Kandhavelu et al., 2017), in which all the proteins of the alternative pathway of complement have been identified in keratitis patient's tear. It has been reported previously that in the case of control open eye-tear and closed-eye tear, some proteins of the complement pathway are present but at a very low level (Willcox et al., 1997). Our data showing the presence of a low level of some of the complement proteins in control reflex tear supports this finding. Among the proteins found in control reflex tear, complement factor C4, C3, CFB, vitronectin, and lactoferrin have also been demonstrated in normal person open-eye tear and closed-eye tear in a previous study (Willcox et al., 1997). However, in keratitis patient tear, these and other alternative complement pathway proteins were upregulated considerably resulting in the representation of the entire alternative pathway of the complement system.

Complement pathways contribute significantly to the innate immune defense against fungal infections by activating host inflammatory response leading to the fungal clearing (Sturtevant and Latgé, 1992b; Morgan and Gasque, 1997; Herbert et al., 2015; Lubbers et al., 2017). Even though the membrane attack complex-mediated lysis of fungal pathogens was not demonstrated, data presented in this study show that an active membrane attack complex in keratitis tear. Ghosh et al. (2015) showed that the low-density membrane attack complexes are involved in signaling. The tear film has a lower level of complement proteins compared to blood. The tear film also has negative regulators such as CFH, clusterin, vitronectin and lactoferrin and these may be reducing the complement activity.

C3 convertase, the alternate pathway convertase, is central to the elaboration of complement function, including formation of C5 convertase. Among the two C3 convertases, the classical pathway C3 convertase, C4b2b is inhibited by lactoferrin (Kievjts and Kijlstra, 1985). Previous reports show the presence of C4 even in control tear, and lactoferrin in tear film can inhibit the classical pathways of complement function (Kandhavelu et al., 2017). There is no change in the amount of lactoferrin in a patient tear compared to the control tear and hence, the role of classical pathway convertases in innate defense in antifungal immunity is minimal or none. Western blot analysis showed increased amount of C3 in a patient tear compared to the control, which has negligible amount of C3 confirming the previous report (Kandhavelu et al., 2017). Under reducing conditions of electrophoresis, alpha chain of purified C3b protein appeared as a 110 kDa band indicating the cleavage of C3a from native C3 alpha chain, which is 120 kDa in molecular weight. Under similar reducing electrophoresis conditions, C3 alpha chain of C3 protein in tear appeared as a 120 kDa protein indicating the absence of cleavage of C3 in a patient tear. Even in blood, very low amount of fluid phase C3b has been demonstrated and hence, it is likely that C3b is not formed in tear film in fluid phase. Examination of the mass spectrometry data from our previous report show the identification of the four peptides (Kandhavelu et al., 2017) belonging to the region covering C3a from patient tear confirming the presence of unprocessed C3 in patient tear.

Spore surface bound forms of C3 and C3 (H_2_O) can in turn bind CFB, which leads to the formation of alternative pathway C3 convertase (Sturtevant and Latgé, 1992a). The presence of factor D and properdin in keratitis patient tear imply that C3bBb convertase could be formed and stabilized on fungal surface. The identification of C3α'1 and C3d in the patient tear shows cleavage of C3b alpha chain presumably by CFH and CFI present in the patient tear film. These results imply the formation of functional MAC complexes, but, their half-life is short due to the cleavage of C3b by CFI. Cleavage of native C3 and C3 (H_2_O) is less effective in the fluid phase, which could be the reason for the presence of uncleaved C3 alpha in patient tear.

In our experiments, pre-incubation of spores with heparin prevents binding of purified C3b and tear C3 to spores. When thiol ester bond is cleaved, any surface with hydroxyl group can bind C3, and hence, it is likely that heparin masks all these sites leading to the inhibition of C3 binding. The actual mechanism of C3b binding to the spore surface is unknown. However, it is interesting to identify ligands that could inhibit C3 binding to spores and such ligands could be used as inhibitors of complement activation and excessive inflammation.

In order to demonstrate the binding of complement proteins of the alternative pathway of complement cascade as well as other proteins that interact with fungal spores, we examined the spectrum of proteins from keratitis tear and healthy person tear films that were bound tightly when incubated with fungal spores. Apart from complement cascade proteins, neutrophil extracellular trap proteins, and inhibitors of MAC were found interacting with fungal spores.

Overall, to the best of our knowledge, this is the first report showing the induction of an active alternative pathway of the complement cascade in keratitis patient's tear. Further, there are other proteins which interact with the fungal spores and these proteins have a significant role to play in the modulation of complement-mediated anti-fungal defense. Induction of the negative regulators implies that the outcome of *A. flavus* infection depends on the host and fungal pathogen interaction in each individual, even though the formation of an active alternative pathway of the complement system is found in all keratitis patients. The identification of swollen conidial surface proteins and their interacting partners in the keratitis patient tear will help in the identification of new virulence mechanisms as well as their role in host immune modulation.

## Data Availability Statement

The raw data supporting the conclusions of this article will be made available by the authors, without undue reservation, to any qualified researcher.

## Ethics Statement

This study was ethically approved from Aravind Eye Hospital Madurai and we have got written informed consent form from all participants in this study.

## Author Contributions

MS performed the experiment and wrote the first draft of manuscript. SK performed hemolysis experiment. RA and JJ guidance and proofreading of manuscript. VN and LP provided tear samples, supervised and reviewed manuscript. DK proposed, designed and supervised the entire study and also, wrote and proofread the manuscript.

## Conflict of Interest

The authors declare that the research was conducted in the absence of any commercial or financial relationships that could be construed as a potential conflict of interest.
